# Supplementary material for: Visualising reaction complexes in amine-based unloaded and CO2-loaded carbon capture solutions
Source: Nat Commun. 2026 Mar 12;17:3828. doi: 10.1038/s41467-026-70391-6 (PMC13121631; doi:10.1038/s41467-026-70391-6)
Supplement: Supplementary file 1 — Supplementary Information [file 41467_2026_70391_MOESM1_ESM.pdf]

Supplementary Information for Visualising Reaction Complexes in Amine Based Unloaded and CO_2 Loaded Carbon Capture Solutions

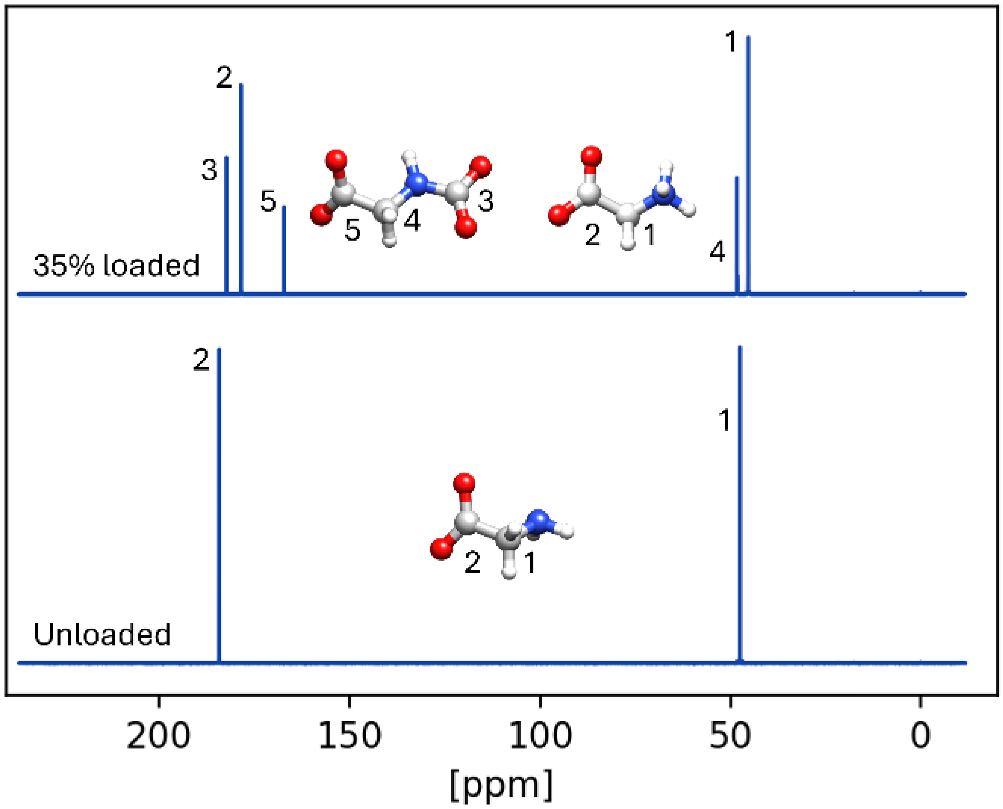

Figure S1. ^{13}C NMR spectra for aqueous K glycinate in unloaded and 35% CO_2 loaded state. At this level of CO_2 loading no bicarbonate signal is observed. All samples were 600 μL in D_2O with 100 μL of 35 mM DSS in D_2O added to each sample to provide a standard and a locking frequency.

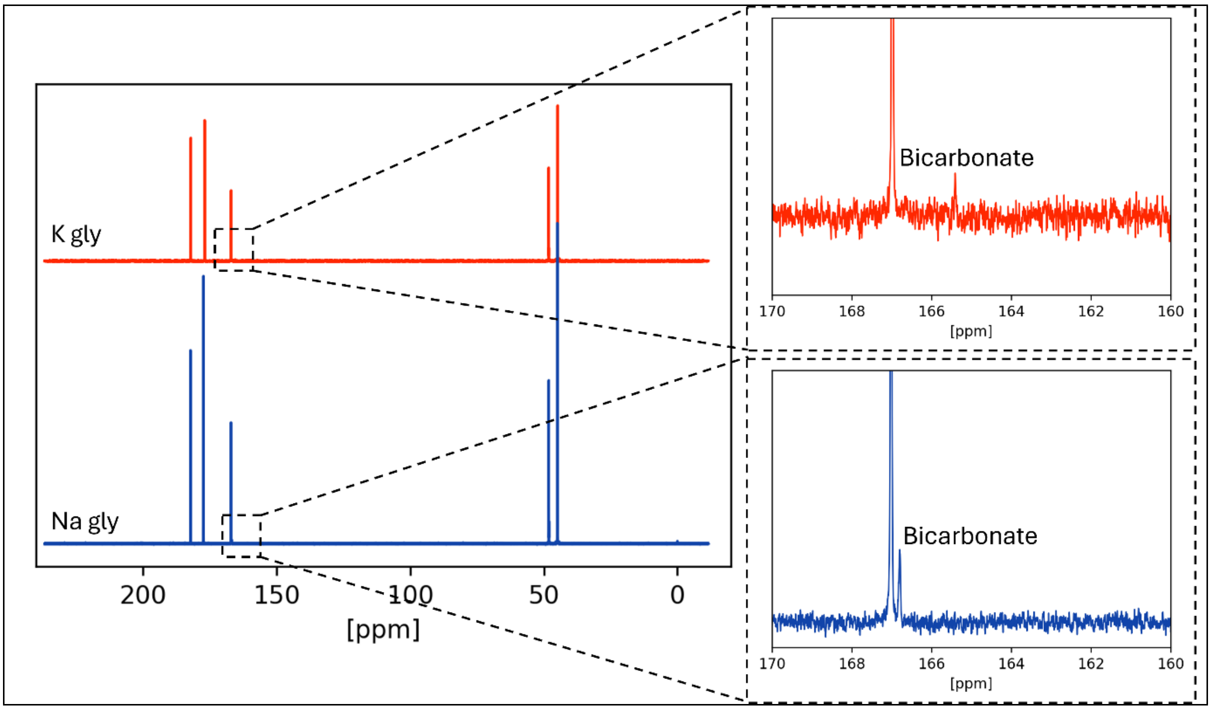

Figure S2. ^{13}C NMR spectra for K glycinate and Na glycinate loaded at 45% respectively using a 400 MHz NMR spectrometer, showing presence of peak corresponding to bicarbonate ion. All samples were 600 µL in D_2O with 100 µL of 35 mM DSS in D_2O added to each sample to provide a standard and a locking frequency.

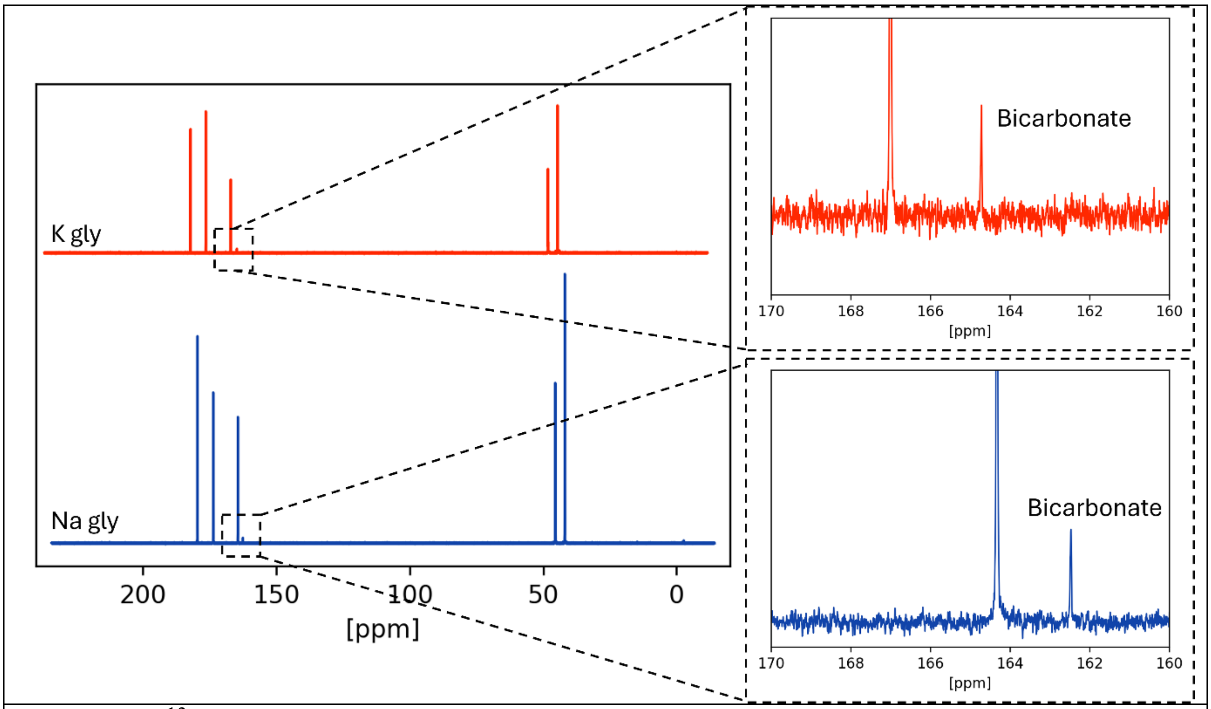

Figure S3. ^{13}C NMR spectra for K glycinate and Na glycinate loaded at 55% respectively using a 400 MHz NMR spectrometer, showing presence of peak corresponding to bicarbonate ion. All samples were 600 µL in D_2O with 100 µL of 35 mM of DSS in D_2O added to each sample to provide a standard and a locking frequency.

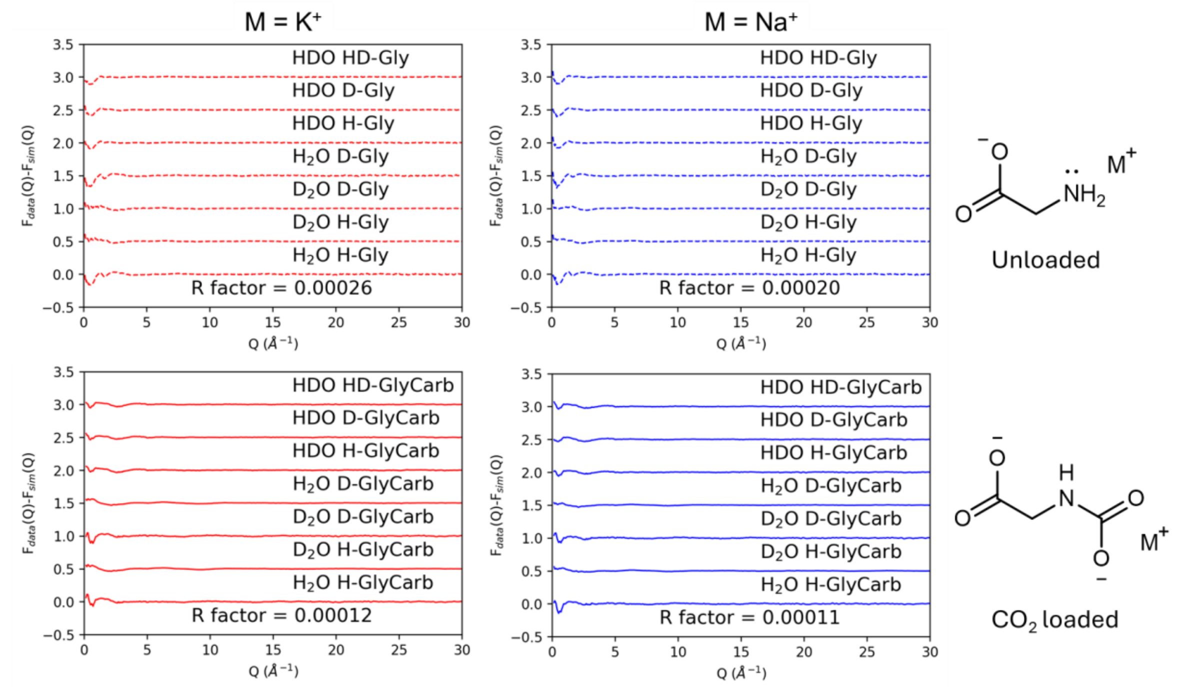

Figure S4. Difference between experimental data and refined stimulation for all relevant datasets. Quality of refined simulation is given by the R factor, show in equation S1, where M is the number of datasets (7 in all instances), $n_Q(i)$ is the number of Q values in the ith dataset, $D_i(Q)$ is the experimental dataset and $F_i(Q)$ is the is the simulated dataset. A perfect overlap between experimental data and refined simulation therefore yields an R factor of 0.

$$R = \frac{1}{M}\sum_i \frac{1}{n_Q(i)}\sum_Q [D_i(Q) - F_i(Q)]^2 \quad \text{(Equation S1)}$$

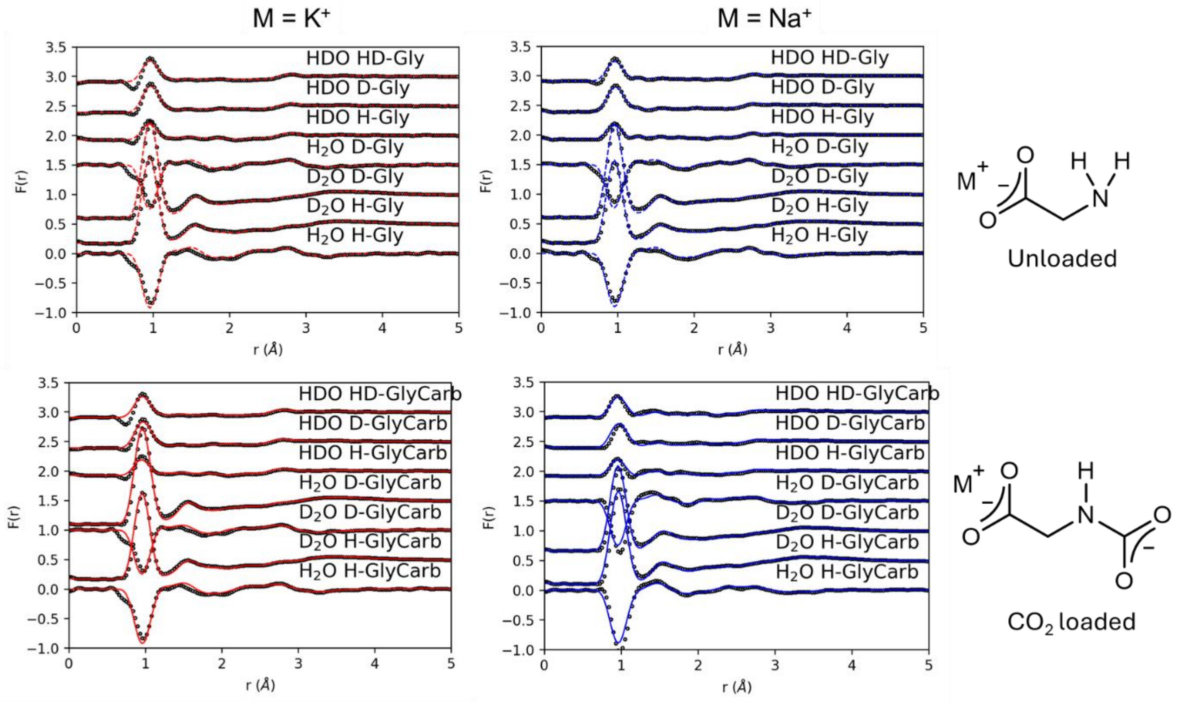

Figure S5. EPSR fits to Fourier transformed experimental neutron diffraction data (open black circles) for 7 H/D isotope variants of K/Na glycinate (red/blue lines) in the unloaded and CO_2 loaded state (dashed/solid lines) to validate intramolecular structures.

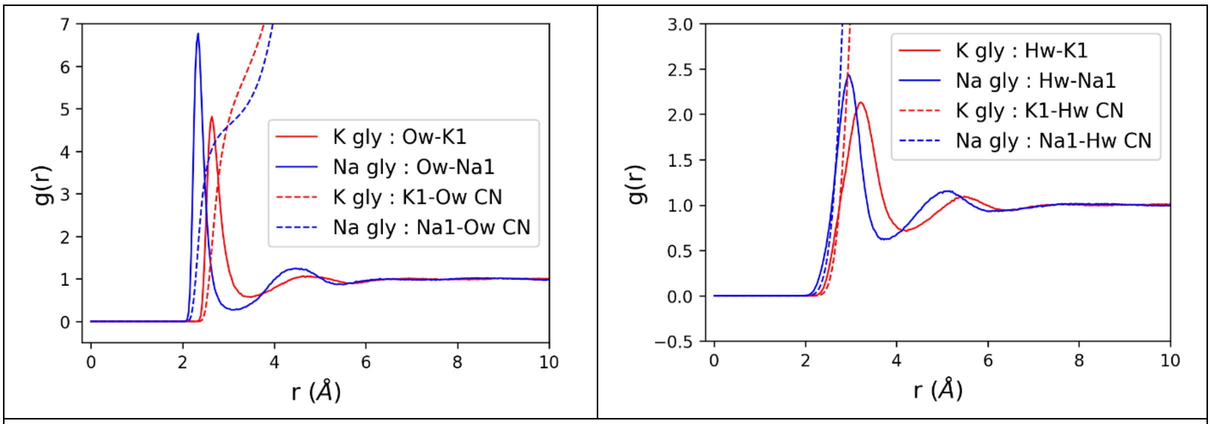

Figure S6. Na^+/K^+-Ow (left) and Na^+/K^+-Hw (right) RDFs for unloaded samples. Presence of higher peaks at shorter distances is indicative of stronger ion – water interactions for Na^+ compared with K^+.

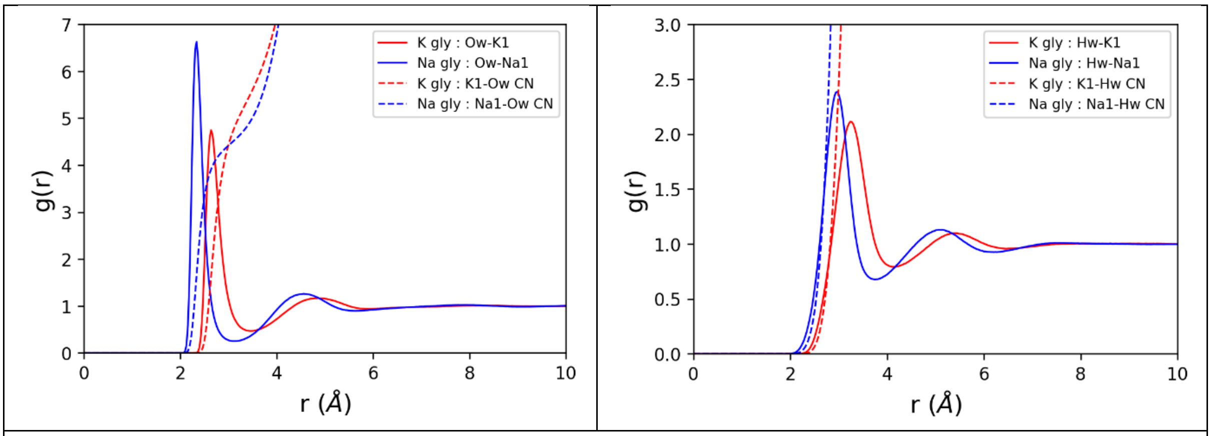

Figure S7. Na^+/K^+-Ow (left) and Na^+/K^+-Hw (right) RDFs for loaded samples. Presence of higher peaks at shorter distances is indicative of stronger ion – water interactions for Na^+ compared with K^+.

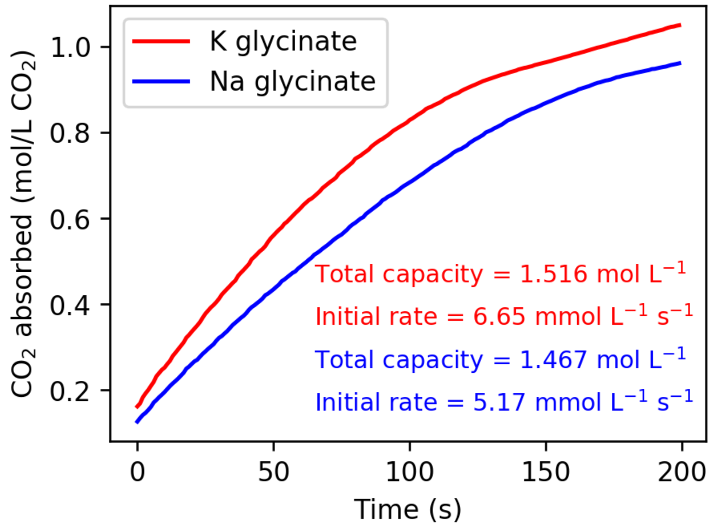

Figure S8. Vapour-liquid equilibrium data for K glycinate and Na glycinate at 2.17 mol salt/kg H_2O.

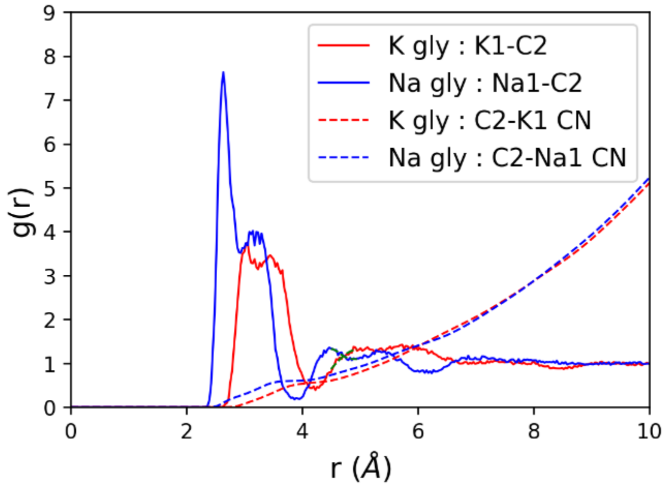

Figure S9. Na^+/K^+- C2a RDFs for unloaded samples. Presence of higher peaks at shorter distances is indicative of stronger ion – water interactions for Na^+ compared with K^+.

**Feature**	**K glycinate**	**Na glycinate**
Modal nearest neighbour $C_{ion}M^+$ distance (Å)	3.04	2.63
$C_{N_{ion}M^+}(4.05\ \text{Å})$	0.54	0.61
$E_{EPSR,N_{ion}M^+}$ (kJ/mol)	-408 ± 2	-479 ± 7

Table S1. Key structural observations in aqueous K glycinate and Na glycinate determined through neutron diffraction and structural refinement. Modal nearest neighbour distances correspond to locations of first peaks in relevant $g_{\alpha\beta}(r)$s. Errors on reported energies correspond to fitting error of location of gaussian peak to calculated EPSR-derived pair interaction energy distributions.

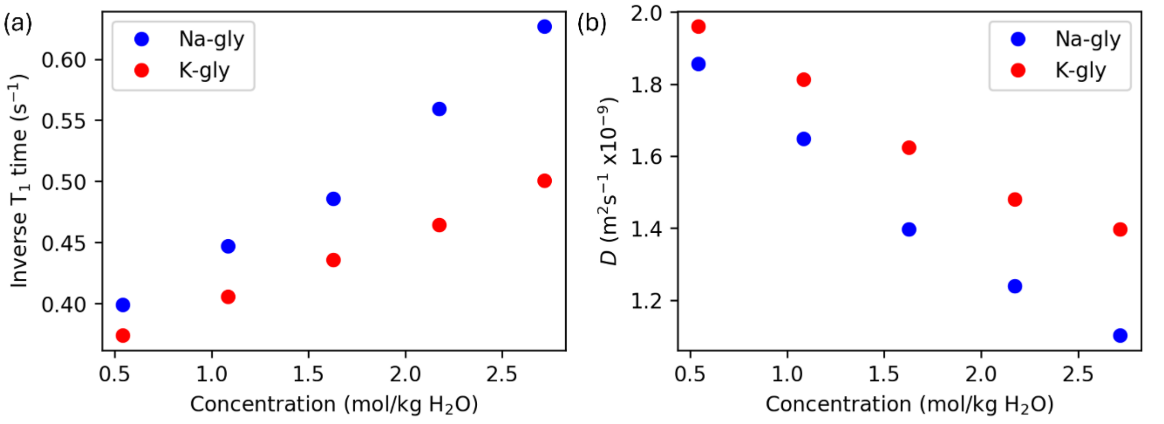

Figure S10. (a) Inverse T_1 decay time, which is proportional to the rotational correlation time of the water molecules[1] (b) Diffusion coefficient of the water molecules as measured through pulsed field gradient spin-echo NMR. These data demonstrate that water molecules in aqueous K glycinate exhibit both faster rotational and diffusive motions compared with in Na glycinate.

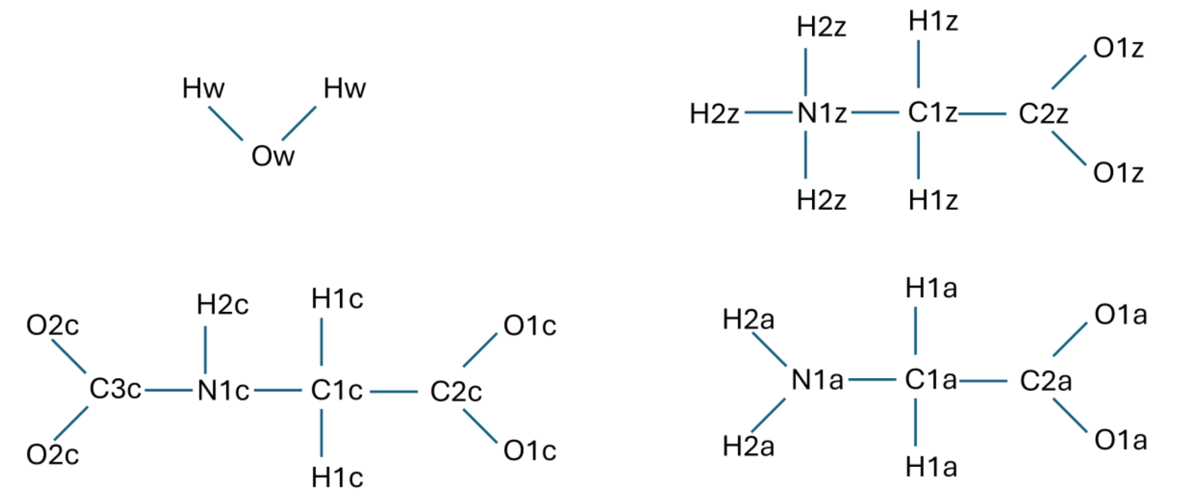

Figure S11. EPSR naming conventions and molecular structures for water (top left), zwitterionic glycine (top right), CO_2 loaded glycine carbamate (bottom left), glycinate anion (bottom right).

Atom	*ε* (kJ/mol)	*σ* (Å)	*q* (e)
Ow	-0.65	3.166	-0.8476
Hw	0	0	0.4238
K	0.5144	2.94	1
Na	0.5144	2.29	1
C1a	0.27614	3.5	-0.0788
C2a	0.29288	3.55	0.4162
H1a	0.1255	2.5	0.0615
N1a	0.71128	3.3	-0.8552
O1a	0.87864	2.96	-0.6209
H2a	0	0	0.3183
C1z	0.4142	3.8	0.09
C2z	0.42932	3.75	0.7
H1z	0	0	0.06
N1z	0.71128	3.25	-0.3
O1z	0.87864	2.96	-0.8
H2z	0	0	0.33
C1c	0.24614	3.5	0.1086
C2c	2.9288	3.55	0.4097
H1c	0.12552	2.5	0.0459
N1c	0.71128	3.25	-1.0357
O1c	0.87864	2.96	-0.6569
H2c	0	0	0.4315
C3c	2.9288	3.55	0.6653
O2c	0.87864	2.96	-0.6787

Table S2. Reference potential parameters employed in the EPSR simulations

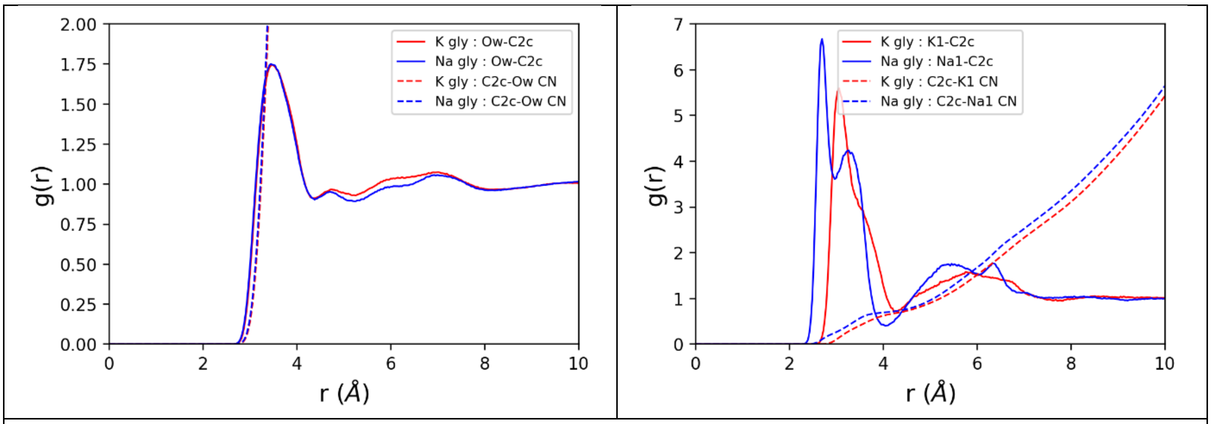

Figure S12. C2c-Ow (left) and C2c-Na^+/K^+ (right) RDFs for loaded samples.

Feature	C2c centred (K glycine carbamate)	C2c centred (Na glycine carbamate)	C3c centred (K glycine carbamate)	C3c centred (Na glycine carbamate)
First peak position in $g_{CO_w}(r)$ (Å)	3.49	3.48	3.40	3.41
C-Ow coordination number over 4.20 Å	8.19	8.36	8.53	8.81
Carboxyl-water EPSR-derived pair interaction energetic interaction (kJ/mol)	-26.0 ± 0.2	-23.0 ± 0.3	-29.4 ± 0.2	-28.3 ± 0.3
First peak position in $g_{C\,cation}(r)$ (Å)	3.06	2.70	3.06	2.72
C-cation coordination number over 4.05 Å	0.63	0.69	0.71	0.68
Carboxyl-cation EPSR-derived pair interaction energetic interaction (kJ/mol)	-407 ± 2	-459 ± 2	-420 ± 2	-487 ± 1
First peak position in $g_{C\,N1z}(r)$ (Å)	3.67	3.32	3.31	3.75
C-glycine zwitterion amine	0.07	0.17	0.07	0.14

coordination number over 4.38 Å				
Carboxyl-zwitterion amine EPSR-derived pair interaction energetic interaction (kJ/mol)	-96 ± 2	-134 ± 3	-165 ± 1	-106 ± 3

Table S3. Key structural observations in aqueous loaded K glycinate and Na glycinate around both carboxyl groups determined through neutron diffraction and structural refinement.

Value	K glycinate	Na glycinate
Unloaded $N_{N_{ion}O_w} \times \Delta H_{N_{ion}O_w}$ (kJ/mol)	-99.1	-94.6
Unloaded $N_{N_{ion}M^+} \times \Delta H_{N_{ion}M^+}$ (kJ/mol)	-44.5	-51.4
Loaded $N_{C_{carb}O_w} \times \Delta H_{C_{carb}O_w}$ (kJ/mol)	-296.9	-294.3
Loaded $N_{C_{carb}M^+} \times \Delta H_{C_{carb}M^+}$ (kJ/mol)	-310.8	-340.9
Loaded $N_{C_{carb}N_{zwit}} \times \Delta H_{C_{carb}N_{zwit}}$ (kJ/mol)	-11.5	-14.8

Table S4. Products of coordination numbers and interaction EPSR-derived pair interaction energies taken from main text tables 1 and 2 to estimate overall EPSR-derived pair interaction energetic parameters for distinct interatomic correlations.

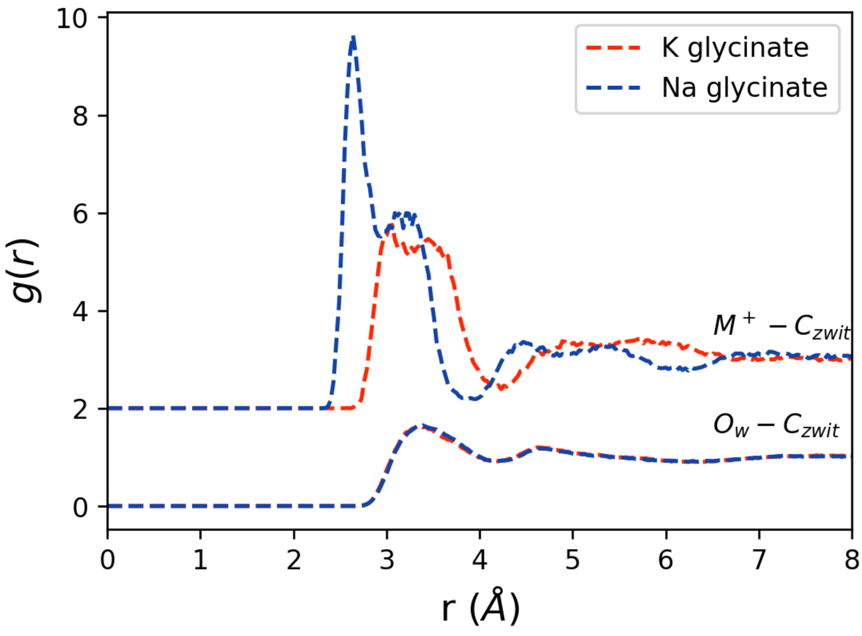

Figure S13. Radial distribution functions for key structural features of interest in aqueous unloaded K/Na glycinate (red/blue dashed lines) centred around carboxylate carbon (C_{ion}).

Sample name	H_2O:D_2O molar ratio	H_5-glycine:D_5-glycine molar ratio	Metal OH:metal OD molar ratio
H_2O K HGly	1:0	1:0	1:0
H_2O K DGly	1:0	0:1	1:0
D_2O K HGly	0:1	1:0	0:1
D_2O K DGly	0:1	0:1	0:1
HDO K HGly	1:1	1:0	1:1
HDO K DGly	1:1	0:1	1:1
HDO K HDGly	1:1	1:1	1:1
H_2O Na HGly	1:0	1:0	1:0
H_2O Na DGly	1:0	0:1	1:0
D_2O Na HGly	0:1	1:0	1:0
D_2O Na DGly	0:1	0:1	1:0
HDO Na HGly	1:1	1:0	1:0
HDO Na DGly	1:1	0:1	1:0
HDO Na HDGly	1:1	1:1	1:0

Table S5. List of isotopic variants studied by neutron scattering

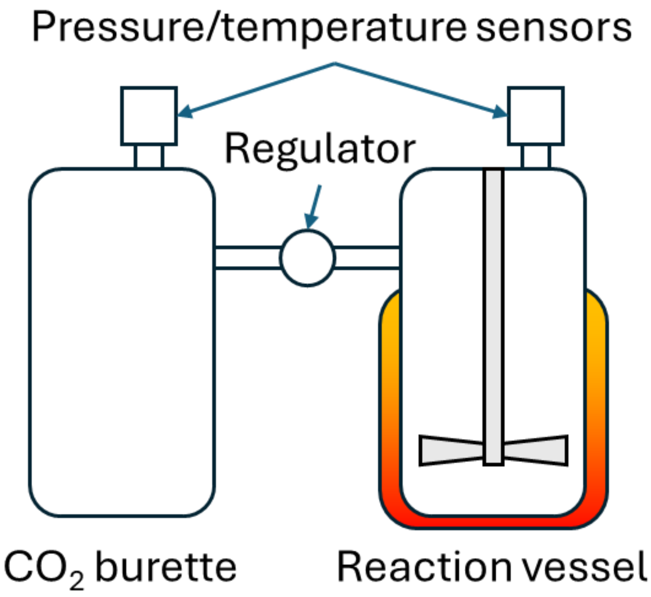

Figure S14. Diagram of custom apparatus for generation of VLE data. Molar absorption of CO_2 is calculated by monitoring both the pressure and temperature of the burette and reaction vessel, as described in the materials and methods section. Determining the molar volume (ν) and therefore the number of moles of CO_2 absorbed is obtained by solving the Beattie-Bridgeman equation. However, this cannot be achieved algebraically and must be done in an iterative method. This iteration is undertaken through a Macro, written in Microsoft Excel and included in the data repository, and trials possible values of ν within a sensible range to produce a value for P. This calculated value is then compared to the measured value of P and the residual used to guide the next input for ν. Once this is calculated, it is then trivial to calculate the molar quantity of absorbed CO_2. This manifests as the difference in total CO_2 transferred from the burette and the total CO_2 present in the headspace of the vessel after equilibrium has been achieved.

Unloaded K glycinate	0.1016
Unloaded Na glycinate	0.1030
CO_2 loaded K glycinate	0.1005
CO_2 loaded Na glycinate	0.1023

Table S6. Sample densities measured in atoms/Å^3

Atom pair	Cutoff – unloaded K gly (Å)	Cutoff – unloaded Na gly (Å)	Cuttof – loaded K gly (Å)	Cutoff – loaded Na gly (Å)
Ow-Ow	3.29	3.29	3.29	3.29
Ow-Hw	2.44	2.44	2.44	2.44
Ow-cation	3.47	3.14	3.47	3.14
Ow-C2a	4.20	4.20	4.20	4.20
Ow-H1a	3.91	3.91	3.91	3.91
Ow-N1a	4.07	4.07	4.07	4.07
Ow-O1a	3.45	3.45	3.45	3.45
Ow-H2a	4.26	4.26	4.26	4.26

Ow-N1z	N/A	N/A	3.43	3.43
Ow-H1z	N/A	N/A	4.12	4.12
Ow-C2z	N/A	N/A	4.53	4.53
Ow-H2z	N/A	N/A	2.42	2.42
Ow-O1z	N/A	N/A	3.12	3.12
Ow-C2c	N/A	N/A	4.51	4.51
Ow-H1c	N/A	N/A	3.71	3.71
Ow-N1c	N/A	N/A	3.53	3.53
Ow-O1c	N/A	N/A	3.39	3.39
Ow-H2c	N/A	N/A	3.81	3.81
Ow-C3c	N/A	N/A	4.51	4.51
Ow-O2c	N/A	N/A	3.25	3.25
N1a-cation	3.40	2.92	3.40	2.92
C2c-cation	4.22	3.89	4.39	4.01
C3c-cation	N/A	N/A	4.39	4.01
C2c-N1z	N/A	N/A	4.45	4.45
C3c-N1z	N/A	N/A	4.45	4.45

Table S7. Cutoff distances employed in EPSR-derived pair interaction energetic calculations for each of the EPSR simulations. Atom naming conventions displayed in figure S4.

Note S1:

To evaluate the dependence of the EPSR-derived pair interaction energies on their cutoff distances we took the approach of varying the cutoff distances by ±10% and recalculating all values. The relative absolute change for the two determined values for each interaction following cutoff distance increase or decrease were then averaged and normalised to the 10% distance variation. This final parameter was assigned a variable δ, and represents how much a particular EPSR-derived pair interaction energy is likely to vary given a relative change in cutoff distance, e.g. a δ value of 1 means that if one varies the cutoff distance by 10%, the EPSR-derived pair interaction energy will also vary by 10%. This approach demonstrated that the more stable the interaction, such as glycinate/glycine carbamate – metal cation interactions, yield low δ values (~0.1), and hence are insensitive findings to cutoff distances, but weaker interactions, such as hydration of the glycinate/glycine carbamate groups, are more variable (~1.6). Full results can be found in supplementary table S8. In all instances however the comparison between whether a particular interaction was more stable in the case of loaded/unloaded K glycinate vs loaded/unloaded Na glycinate when using identical cutoff distances were unchanged. In a similar fashion, the sensitivity of the coordination numbers to the employed calculation distance was also evaluated by varying the cutoff distances by ±0.06 Å and recalculating all values. This distance was chosen as the coordination number and $g_{\alpha\beta}(r)$ data was binned in 0.03 Å increments. More stable interactions, regardless of how infrequent they may be, such as glycinate amine – metal cation interactions, were less variable than less stable interactions. Full results can be found in supplementary tables S9 and S10. We derived an uncertainty for the reported coordination numbers using a bootstrapping approach, where the simulations were allowed to accumulate statistics over short runs (100 iterations),

and the relevant coordination numbers were calculated. This was repeated 10 times to derive an average coordination number and an associated standard error. In all instances these errors were unresolvable to 2 decimal places, and as such have not been reported here. The full results of this approach can be found in supplementary tables S9 and S10.

The sensitivity of the final results to the employed reference potentials is important to evaluate. This is likely to be particularly important for samples that contain several different species, or species that exist at relatively low concentration, as these will have lower contributions to the overall experimental scattering data, and therefore structural refinement will be less effective at resolving their interatomic correlations accurately[2]. With this in mind, we employed four different sets of reference potentials for the most complex samples studied here, the CO_2 loaded K/Na glycinate solutions, to evaluate their influence. These were: (1) the 'base' reference potentials described in supplementary table S1, derived using a combination of LigParGen[3] and previous data[4–7] and which gave the lowest R factor following potential refinement, (2) a set where the glycinate anion force field was substituted for a LigParGen generated alternative, (3) a set where the zwitterionic glycine was substituted for a LigParGen generated alternative, (4) a set where the metal cation force field was substituted for that described by Loche *et al*[8]. These forcefields are detailed in supplementary tables S11-13. The water force field was not varied as it is strongly weighted in the data, and hence is the least likely to be impacted by force field variation, and commonly employed water reference potentials (SPC, SPC/E, TIP3P) are all observed to be very similar[9], and the carbamate force field was not varied as this was already generated using LigParGen as a sensible alternative is not available. Using these four different reference potential sets, potential refinement was performed as described and the various $g_{\alpha\beta}(r)$s were produced. For each interaction of interest, the four different $g_{\alpha\beta}(r)$s were arithmetically averaged to generate an 'average' $\overline{g}_{\alpha\beta}(r)$, and their variability quantified using the same approach as is used to determine the 'R factor' employed in structural refinement, as described in equation S2, and assigned the variable Σ. Four identical $g_{\alpha\beta}(r)$s would therefore produce a value of 0. To estimate an upper limit for Σ, we deliberately compared four different $g_{\alpha\beta}(r)$s, one from each of the four reference potential variations for the CO_2 loaded K glycinate data (O_wO_w, K^+C_{ion}, $N_{zwit}C_{zwit}$, H_wN_{carb}). These produce a value Σ_0 of 0.0373. The resultant $g_{\alpha\beta}(r)$s are shown in supplementary figure S15. As expected, the more strongly weighted a particular interatomic correlation is in the experimental scattering data, due to the combined influence of its coherent scattering length and molar abundance, and frequency of interaction (quantified through coordination numbers), the lower the value of Σ. We therefore observe that correlations involving water are robust ($\Sigma \sim 0.0005$), however correlations involving glycinate/glycine carbamate, and metal cations are more variable ($\Sigma \sim 0.02$), particularly those that are infrequently occurring, such as glycine carbamate – glycine zwitterion interactions ($\Sigma \sim 0.035$). The full results are detailed supplementary table S14. Overall, considering the intermolecular correlations we predict to be dominant by weighting their EPSR-derived pair interaction energies by their coordination numbers, the findings of this work are deemed to be robust. It is important to note at this point that the results deriving from any of these forcefields should not be considered incorrect, as all are sensible starting

parameters, merely that for some correlations EPSR is less able to offer a single unique solution than others.'

Interaction	δ - K glycinate	δ - Na glycinate
Unloaded N1a-Ow	1.31	1.58
Unloaded N1a-M^+	0.10	0.04
Unloaded C2a-M^+	0.04	0.13
Loaded bulk water-water hydrogen bonding	1.20	1.20
Unloaded N1a-N1a	N/A	N/A
Loaded C3c-Ow	1.82	1.80
Loaded C3c-M^+	0.25	0.07
Loaded bulk water-water hydrogen bonding	1.51	1.50
Loaded C3c-N1z	0.40	0.95

Table S8. Determined values for variability δ of EPSR-derived pair interaction energies dependent on the employed cutoff distances for discussed interatomic correlations in the main text.

Interaction (K systems)	Distance (Å)	CN (at distance)	CN (distance - 0.06 Å)	CN (distance + 0.06 Å)	Error from bootstrapping (%)
Unloaded N1a-Ow	4.08	7.05	6.70	7.41	0.157
Unloaded N1a-M^+	3.15	0.14	0.13	0.14	2.59
Unloaded C2a-M^+	4.05	0.54	0.53	0.55	0.374
Unloaded Ow-Ow	3.30	3.86	3.65	4.07	0.0165
Unloaded N1a-N1a	4.14	0.17	0.16	0.18	3.45
Loaded C3c-Ow	4.50	10.1	9.8	10.46	0.234
Loaded C3c-M^+	4.20	0.74	0.73	0.75	0.834
Loaded Ow-Ow	3.30	3.76	3.51	4.02	0.051
Loaded C3c-N1z	4.38	0.07	0.06	0.07	4.638

Table S9. Raw data used to determine percentage errors in coordination numbers in K glycinate systems through a bootstrapping approach, where 10 runs of 100 accumulated simulation iterations were averaged to determine a standard error.

Interaction (K systems)	Distance (Å)	CN (at distance)	CN (distance - 0.06 Å)	CN (distance + 0.06 Å)	Error from bootstrapping (%)
Unloaded N1a-Ow	4.08	7.05	6.70	7.41	0.181

Unloaded N1a-M^+	3.15	0.14	0.13	0.14	0.464
Unloaded C2a-M^+	4.05	0.54	0.53	0.55	0.339
Unloaded Ow-Ow	3.30	3.86	3.65	4.07	0.039
Unloaded N1a-N1a	4.14	0.17	0.16	0.18	2.15
Loaded C3c-Ow	4.50	10.1	9.8	10.46	0.190
Loaded C3c-M^+	4.20	0.74	0.73	0.75	0.671
Loaded Ow-Ow	3.30	3.76	3.51	4.02	0.079
Loaded C3c-N1z	4.38	0.07	0.06	0.07	0.690

Table S10. Raw data used to determine percentage errors in coordination numbers in Na glycinate systems through a bootstrapping approach, where 10 runs of 100 accumulated simulation iterations were averaged to determine a standard error.

Atom	ε (kJ/mol)	σ (Å)	q (e)
C1a	0.276144	3.5	-0.0601
C2a	0.29288	3.55	0.4248
H1a	0.12552	2.5	0.0478
N1a	0.71128	3.3	-0.7946
O1a	0.87864	2.96	-0.6209
H2a	0	0	0.288

Table S11. Alternative potential parameters for glycinate anion derived from LigParGen

Atom	ε (kJ/mol)	σ (Å)	q (e)
C1z	0.276144	3.5	-0.234
C2z	0.29288	3.55	0.482
H1z	0.12552	2.5	0.1343
N1z	0.71128	3.25	-0.3587
O1z	0.87864	2.96	-0.6304
H2z	0	0	0.3676

Table S12. Alternative potential parameters for glycine zwitterion derived from LigParGen

Atom	ε (kJ/mol)	σ (Å)	q (e)
K	0.9	2.83	1
Na	0.45	0.231	1

Table S13. Alternative potential parameters for metal cations derived from Loche *et al*[8]

$$\Sigma = \frac{1}{M}\sum_{i}\frac{1}{n_r(i)}\sum_{Q}\left[g_{\alpha\beta,i}(r) - \overline{g}_{\alpha\beta}(r)\right]^2 \quad \text{(Equation S2)}$$

Interaction	Σ - K glycinate	Σ - Na glycinate
Loaded C3c-Ow	0.0003	0.0003

Loaded C3c-M^+	0.0191	0.0197
Loaded Ow-Ow	0.0008	0.0007
Loaded C3c-N1z	0.0374	0.0311

Table S14. Determined values for the variability of the CO_2 loaded $g_{\alpha\beta}(r)$s discussed in the main text resulting from changing the starting reference potentials.

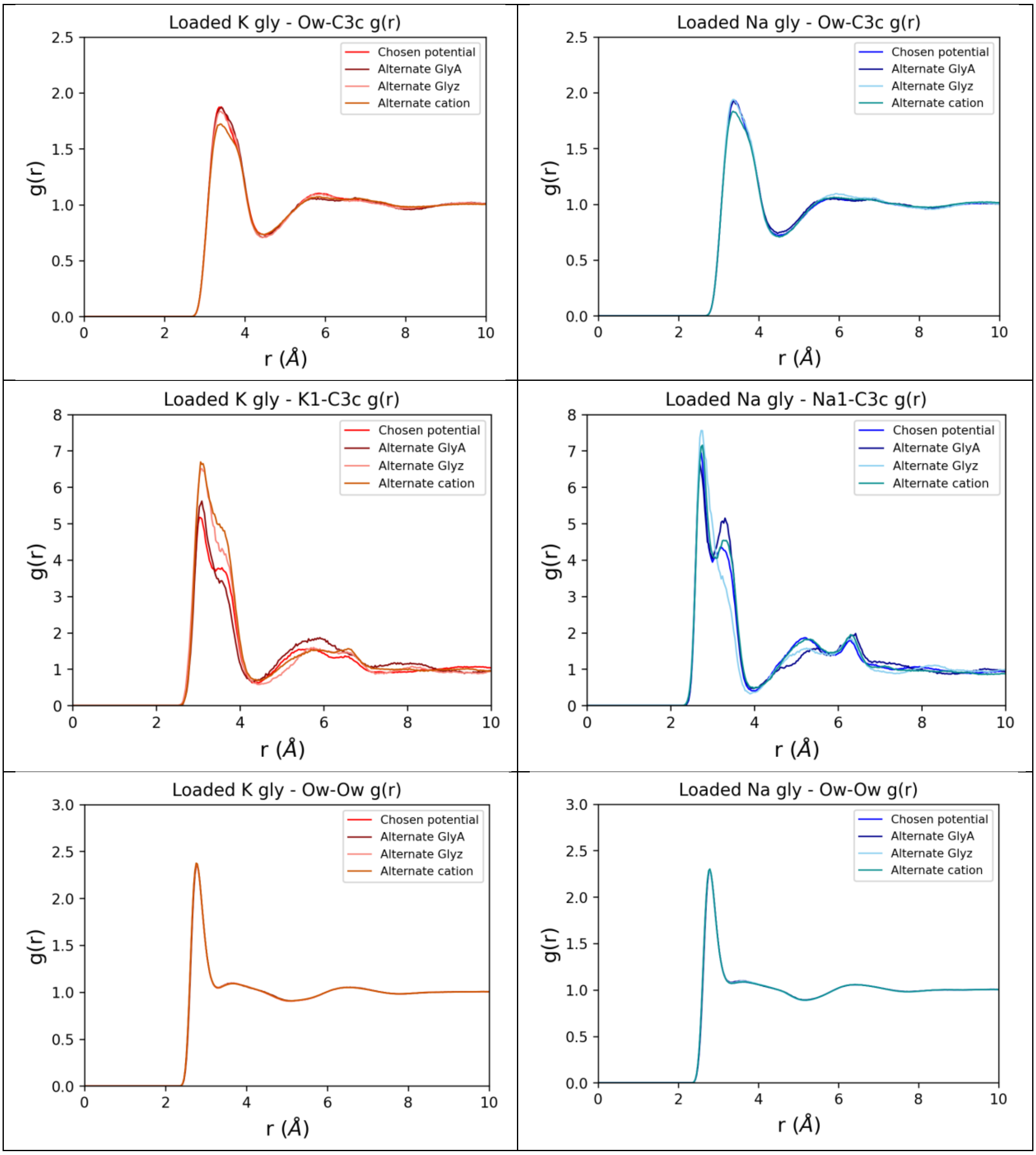

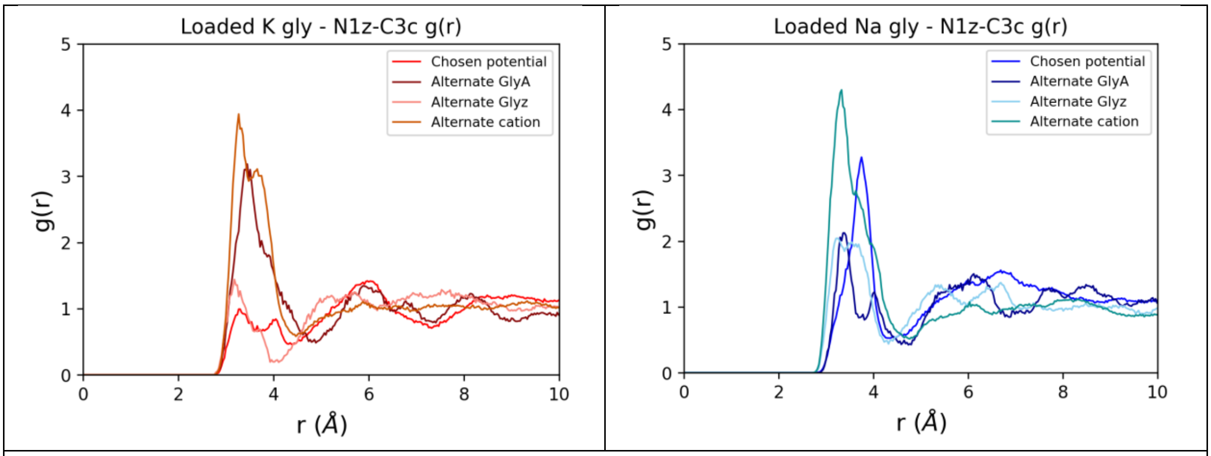

Figure S15. Resultant radial distribution functions following structural refinement resulting from varying the reference potentials as described through tables S2, S11, S12, and S13.

+

Supplementary References

1. Engel, G. & Hertz, H. On the Negative Hydration. A Nuclear Magnetic Relaxation Study. *Berichte der Bunsengesellschaft für Phys. Chemie* **72**, 808–834 (1968).
2. Soper, A. K. Empirical potential Monte Carlo simulation of fluid structure. *Chem. Phys.* **202**, 295–306 (1996).
3. Dodda, L. S., De Vaca, I. C., Tirado-Rives, J. & Jorgensen, W. L. LigParGen web server: An automatic OPLS-AA parameter generator for organic ligands. *Nucleic Acids Res.* **45**, W331–W336 (2017).
4. Laurent, H., Soper, A. & Dougan, L. Biomolecular self-assembly under extreme Martian mimetic conditions. *Mol. Phys.* **117**, 3398–3407 (2019).
5. Di Gioacchino, M., Ricci, M. A., Imberti, S., Holzmann, N. & Bruni, F. Hydration and aggregation of a simple amino acid: The case of glycine. *J. Mol. Liq.* **301**, 112407 (2020).
6. Sweatman, M. B., Afify, N. D., Ferreiro-Rangel, C. A., Jorge, M. & Sefcik, J. Molecular Dynamics Investigation of Clustering in Aqueous Glycine Solutions. *J. Phys. Chem. B* **126**, 4711–4722 (2022).
7. Mancinelli, R., Botti, A., Bruni, F., Ricci, M. A. & Soper, A. K. Perturbation of water structure due to monovalent ions in solution. *Phys. Chem. Chem. Phys.* **9**, 2959–2967 (2007).
8. Loche, P., Steinbrunner, P., Friedowitz, S., Netz, R. R. & Bonthuis, D. J. Transferable Ion Force Fields in Water from a Simultaneous Optimization of Ion Solvation and Ion-Ion Interaction. *J. Phys. Chem. B* **125**, 8581–8587 (2021).
9. Mark, P. & Nilsson, L. Structure and dynamics of the TIP3P, SPC, and SPC/E water models at 298 K. *J. Phys. Chem. A* **105**, 9954–9960 (2001).
